# Reversible Posterior Leukoencephalopathy Syndrome Developing After Restart of Sunitinib Therapy for Metastatic Renal Cell Carcinoma

**DOI:** 10.1155/2016/6852951

**Published:** 2016-10-04

**Authors:** Shinji Fukui, Yuta Toyoshima, Takeshi Inoue, Yoriaki Kagebayashi, Shoji Samma

**Affiliations:** Department of Urology, Nara Prefecture General Medical Center, 1-30-1 Hiramatsu, Nara, Nara 631-0846, Japan

## Abstract

A 64-year-old Japanese man had started molecular-targeted therapy with sunitinib for lymph node metastasis 5 years after nephrectomy for left renal cell carcinoma (clear cell carcinoma, G2, pT2N0M0). He was transported to our emergency department because of generalized tonic-clonic seizure, vision loss, and impaired consciousness with acute hypertension after 8 cycles of treatment (2 years after the initiation of sunitinib therapy, including a drug withdrawal period for one year). MRI of the brain (FLAIR images) showed multiple high-intensity lesions in the white matter of the occipital and cerebellar lobes, dorsal brain stem, and left thalamus. Reversible posterior leukoencephalopathy syndrome caused by sunitinib was suspected. In addition to the immediate discontinuation of sunitinib therapy, the administration of antihypertensive agents and anticonvulsants improved the clinical symptoms without neurological damage. Physicians should be aware that sunitinib causes reversible posterior leukoencephalopathy syndrome. The early recognition of reversible posterior leukoencephalopathy syndrome is critical to avoid irreversible neurological damage.

## 1. Introduction

Kidney tumors comprise approximately 2% of all malignancies in adults [1], and the majority of kidney tumors are renal cell carcinomas (RCCs). Approximately 30% of RCCs involve metastatic disease at the diagnosis, and 50% of patients who receive curative surgery experience RCC relapse at distant sites [2]. Sunitinib is one of the tyrosine kinase inhibitors. It has been elucidated that sunitinib inhibits vascular endothelial growth factor receptors and platelet-derived growth factor receptors, subsequently leading to the inhibition of tumor angiogenesis. Sunitinib was approved for advanced RCC in 2006 in Japan. Well-known adverse events caused by sunitinib include hypertension, fatigue, thyroid dysfunction, cardiotoxicity, gastrointestinal toxicities such as diarrhea, stomatitis and nausea, leukocytopenia, thrombocytopenia, and skin toxicity. Herein, we describe the first reported Japanese case of reversible posterior leukoencephalopathy syndrome (RPLS) that developed on receiving sunitinib therapy for metastatic RCC.

## 2. Case Report

A 64-year-old man had undergone left nephrectomy for left renal cell carcinoma (clear cell carcinoma, G2, pT2N0M0) at the age of 57 years. Treatment with sunitinib (50 mg daily for 4 weeks every 6 weeks) was initiated for the metastasis of stomach greater curvature lymph nodes 5 years after left nephrectomy. Physical examination demonstrated no relevant abnormalities. His performance status was 0, and the blood pressure was 120/80 mmHg. Mild renal insufficiency (the serum creatinine level was 1.26 mg/dL, and the estimated glomerular filtration rate was 45.7 mL/min) was noted on laboratory examination. Sunitinib treatment proceeded well without deterioration of the renal function, elevation of the blood pressure, or thyroid dysfunction. Computed tomography (CT) demonstrated stable disease after 4 cycles of sunitinib treatment. He requested the discontinuation of sunitinib treatment because of general fatigue, and he was followed up without sunitinib administration. Treatment with sunitinib was restarted (50 mg daily for 4 weeks every 6 weeks) one year after its discontinuation because of progressive disease (new metastatic lesions on the left adrenal gland and pancreas head) on enhanced CT.

He was transported to our emergency department with symptoms of generalized tonic-clonic seizure, vision loss, and lack of consciousness 5 months after restarting the sunitinib therapy (2 years since the initiation of sunitinib therapy). The symptoms developed during an on-period (day 23) of the sunitinib therapy. He had no history of head injury, epilepsy, or hypertension, nor a family history of neurological or psychiatric disorders.

**Figure 1:**
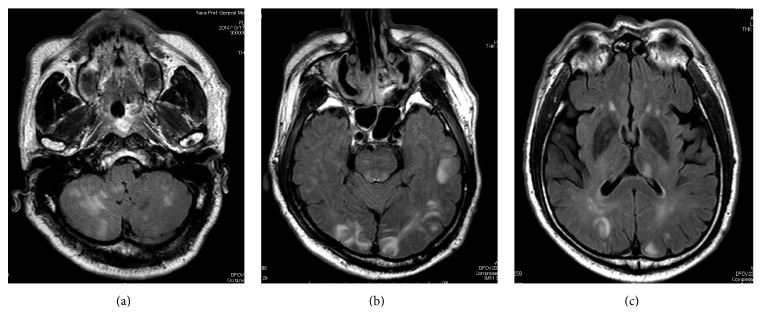
MRI (FLAIR images) of the brain on admission. Multiple high-intensity lesions in the white matter of the occipital and cerebellar lobes, dorsal brain stem, and left thalamus were demonstrated, suggesting RPLS. ((a) Cerebellar lobes lesions, (b) occipital lobes and dorsal brain stem lesions, and (c) left thalamus and occipital lobes lesions.)

**Figure 2:**
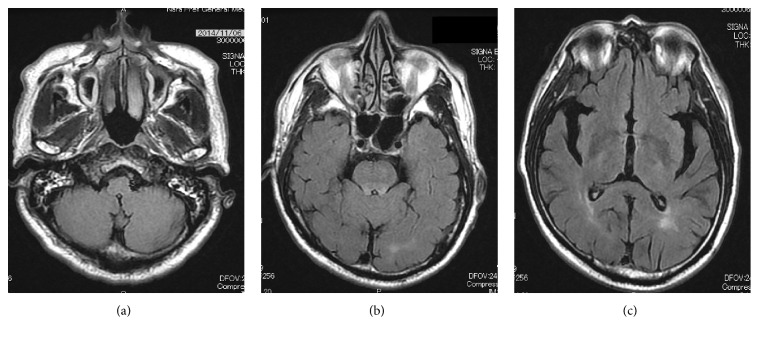
MRI (FLAIR images) of the brain after treatment. The multiple high-intensity lesions significantly improved. ((a) Cerebellar lobes lesions, (b) occipital lobes and dorsal brain stem lesions, and (c) left thalamus and occipital lobes lesions.)

He was afebrile, and his pupillary reflexes were prompt and preserved bilaterally. The blood pressure was markedly elevated at the time of presentation (230/129 mmHg). Blood examination suggested no electrolyte imbalance. Brain CT demonstrated no evidence of metastatic brain tumors nor intracerebral hemorrhage. Magnetic resonance imaging (MRI) of the brain (FLAIR images) showed multiple high-intensity lesions in the white matter of the occipital and cerebellar lobes, dorsal brain stem, and left thalamus (Figure 1). Diffusion-weighted imaging (DWI) on MRI showed the elevation of ADC value mapping. According to the MRI findings and clinical course, RPLS caused by sunitinib treatment was suspected. Sunitinib was discontinued immediately. An antihypertensive drug, nicardipine, was started to control the blood pressure, and an anticonvulsant, fosphenytoin, was administered to control seizures. The blood pressure rapidly returned to normal. MRI of the brain 20 days after admission showed significant resolution of the multiple high-intensity lesions in the white matter (Figure 2). No episode of seizure developed during the hospitalization, and the blood pressure was maintained as normal without any antihypertensive drugs in 3 weeks. He was discharged from the hospital 26 days after admission without any neurological symptoms. Although an m-TOR inhibitor, everolimus, was started for metastatic lesions of the lymph node, left adrenal, and pancreas head, he died of the disease 5 months after administration of the m-TOR inhibitor.

## 3. Discussion

RPLS was first described by Hinchey et al. [9], and typical clinical symptoms of RPLS include headache, seizures, visual abnormalities, acute hypertension, and an altered mental status. RPLS is also referred to as posterior reversible encephalopathy syndrome (PRES). RPLS correlates with cerebral vasogenic edema because of the elevation of the blood pressure, and toxic damage to the blood-brain barrier or vascular endothelium [9]. Its pathogenesis involves the disruption of cerebral vascular endothelial cells and impaired cerebrovascular autoregulation, which lead to cerebral edema secondary to a variety of conditions, including arterial hypertension, eclampsia, collagen vascular disorders, Guillain-Barre Syndrome, and thrombotic thrombocytic purpura [3]. Some immunosuppressive agents and cytotoxic drugs such as tacrolimus, cyclosporin A, and cisplatin [3] lead to cerebral cytotoxic edema and may cause RPLS [9]. RPLS is also accompanied by intracerebral and subarachnoid hemorrhage in 10 to 25% of cases [10].

The typical MRI findings of RPLS are edematous lesions involving the white matter in the posterior portions of the cerebral hemispheres, particularly bilaterally in the parietooccipital regions in 98% of the patients [9, 11, 12], although other areas of the brain such as the frontal lobes (78.9%), temporal lobes (68.4%), cerebellar lobes (34.2%), thalamus (30.3%), and brain stem (18.4%) may also be affected [12]. DWI on MRI shows the elevation of ADC value mapping [13]. In our case, MRI showed multiple high-intensity lesions in the white matter not only of the occipital and cerebellar lobes, but also of the dorsal brain stem and left thalamus, although it is minor involvement, with a high ADC value on DWI, being a typical MRI finding of RPLS.

RPLS has been reported to be caused by treatment with vascular endothelial growth factor receptor-inhibitors such as bevacizumab and sorafenib [3]. There is, however, a very rare entity of RPLS triggered by treatment with sunitinib. According to the database PubMed using the key words of “reversible posterior leukoencephalopathy syndrome and sunitinib”, only 11 cases of RPLS due to sunitinib have been reported in the literature (Table 1) [3–8]. The duration until the onset after the administration of sunitinib varies from several days to several months. Our case showed the longest interval: 2 years since the initial induction, and 5 months since the restart of sunitinib. The blood pressure at the presentation of clinical symptoms of RPLS was elevated in all except one patient. All patients discontinued sunitinib immediately after the onset of RPLS. Nine out of 12 patients received antihypertensive agents to control the blood pressure, and 8 out of the 12 patients received anticonvulsants to manage seizures. In all the patients except one, prompt and appropriate managements completely resolved their neurological symptoms in a few days to several weeks. The remaining one died from RCC in a few weeks after the onset of RPLS. After the complete resolution of symptoms associated with RPLS, all the patients did not restart sunitinib.

In our case, RPLS due to sunitinib occurred after 5-month medication with the drug after a drug withdrawal period of one year. We do not know the mechanism of the development of RPLS after restarting sunitinib therapy, although the patient had no signs of RPLS before the withdrawal of the drug for one year. According to the clinical signs and MRI findings, however, he was diagnosed with RPLS due to sunitinib.

Although MRI findings of RPLS improved in a median of 20 days in 88% of patients [3], the neurological damage could be potentially life-threatening or irreversible if RPLS is not treated immediately, and any causative drugs are not discontinued [14, 15]. Physicians should monitor the patients closely, and the early recognition of RPLS is critical to avoid irreversible neurological damage.

In conclusion, we describe the first reported Japanese case of RPLS due to sunitinib. Although neurological damage of RPLS is generally reversible when managed promptly, physicians should be aware that it may progress to irreversible neurological damage, possibly leading to death.

## Figures and Tables

**Table 1:** Reversible posterior leukoencephalopathy syndrome caused by sunitinib treatment.

Case	Age/gender	Disease	Onset after sunitinib	Sunitinib dose (mg)	Blood pressure (mmHg)	Management	Clinical outcome of RPLS
1 [3]	54/F	GIST	8 months	50	210/110	Sunitinib discontinuation antihypertensive drug anticonvulsant	Complete recovery in 10 days
2 [3]	70/F	RCC	2 weeks	50	170/100	Complete discontinuation antihypertensive drug anticonvulsant	Complete recovery in a few days
3 [3]	81/F	RCC	5 months	—	130/74	Sunitinib discontinuation	Complete recovery in 1 month
4 [4]	84/F	RCC	2 weeks	50	142/72	Sunitinib discontinuation	Complete recovery in 3 days
5 [3]	39/F	RCC	1 week	—	160/102	Sunitinib discontinuation antihypertensive drug anticonvulsant	Complete recovery in 2 weeks
6 [3]	48/F	RCC	1 week	50	190/130	Sunitinib discontinuation	Complete recovery in 3 weeks
7 [3]	65/M	RCC	8 days	50	160/100	Sunitinib discontinuation antihypertensive drug	Complete recovery in 17 days
8 [5]	61/M	RCC	15 weeks	50	202/101	Sunitinib discontinuation antihypertensive drug anticonvulsant	Complete recovery in 10 weeks
9 [6]	48/F	RCC	3 months	50	178/117	Sunitinib discontinuation antihypertensive drug anticonvulsant	Complete recovery in 8 weeks
10 [7]	71/F	RCC	8 months	37.5	179/110	Sunitinib discontinuation antihypertensive drug anticonvulsant	Complete recovery in 3 days
11 [8]	67/M	RCC	2 months	50	180/100	Sunitinib discontinuation antihypertensive drug anticonvulsant	Complete recovery not achieved (cancer death in a few weeks)
Present case	64/M	RCC	2 years (5 months from reinitiation)	50	230/129	Sunitinib discontinuation antihypertensive drug anticonvulsant	Complete recovery in 3 weeks
